# Acute Effects of Combining Weight and Elastic Resistance Exercise on Vascular Function in Older Adults

**DOI:** 10.3390/geriatrics9030056

**Published:** 2024-04-27

**Authors:** Kampanart Paditsaeree, Witid Mitranun

**Affiliations:** 1 Department of Physical Education, Faculty of Education, Phuket Rajabhat University, Phuket 83000, Thailand; kampanatsmart@hotmail.com; 2 Department of Sports Science, Faculty of Physical Education, Sports, and Health, Srinakharinwirot University, Nakhon Nayok 26120, Thailand

**Keywords:** variable resistance, endothelial function, flow-mediated dilation, pulse wave velocity, senior citizen

## Abstract

Prior research has suggested that resistance exercise may result in a temporary decrease in vascular function, as measured by flow-mediated dilation (FMD), among untrained young individuals. However, the immediate impact of combined elastic and weight resistance training on older adults remains insufficiently explored. We assessed vascular function before, after, and 30 min after acute exercise under three resistance conditions to evaluate whether a combination of weight and elastic resistance exercises has an acute effect on vascular function in older adults. Fourteen older adults (65.6 ± 2.9 years) executed three sets of 12 repetitions at 65% of one repetition maximum (1 RM) of the bench press (BP) exercise. Testing was performed on three separate days as follows: (1) barbell alone (BA); (2) barbell plus elastic bands (10% of 65% 1 RM) (BE10); and (3) barbell plus elastic bands (20% of 65% 1 RM) (BE20). A two-way (time × condition) repeated measures analysis of variance was employed to assess the time and condition effects on flow-mediated dilation (FMD) and pulse wave velocity (PWV). At 0 min post-exercise, FMD was significantly higher during BE10 than during BA (*p* < 0.05); however, at 30 min post-exercise, no significant difference (*p* ≥ 0.05) was observed between the three conditions. In each condition, FMD results did not differ significantly at different times (*p* ≥ 0.05). For FMD, the main effect of the condition (F[2,26] = 3.86, *p* = 0.034) and that of the time and condition (F[4,52] = 3.66, *p* = 0.011) were significant. For PWV, only the difference between the BA and BE10 conditions was significant at 0 min (*p* < 0.05). PWV increased from baseline in the BA condition (*p* < 0.05) but not significantly in the BE10 and BE20 conditions (*p* ≥ 0.05). Therefore, BA, BE10, and BE20 demonstrated various changes in vascular function. Long-term training intervention studies are needed to validate these findings.

## 1. Introduction

The endothelium plays a crucial role in controlling systemic blood pressure and vascular tone. The non-pharmacological flow-mediated dilation (FMD) test is a non-invasive method with a high reproducibility rate [1] assessed in in vivo endothelial-dependent vasodilation [2], which results from a response to shear stress and causes the release of nitric oxide (NO), prostacyclin, and endothelial-derived hyperpolarizing factor [3]. Aging can contribute to endothelial dysfunction resulting from mechanical denudation, hemodynamic forces, immune complex deposition, irradiation, and chemicals [4]. Endothelial dysfunction is associated with an increase in pulse wave velocity (PWV), an indicator of arterial stiffness [5], and a reduction in FMD [4]. Both PWV and FMD have been clinically employed as indicators of cardiovascular disease, cerebrovascular disease, congestive heart failure, and mortality risks [6,7,8].

Resistance training for vascular health has been increasingly investigated [9,10,11]. For example, the study by Rakobowchuk et al. [11] demonstrated that after resistance training, FMD did not change, and arterial stiffness increased in young adults. A recent scoping analysis has demonstrated that approximately half of the trials conducted on middle-aged and older adults revealed little or no vascular benefit from resistance exercise [12]. Modifying a resistance program to maintain muscle benefit while improving vascular function may begin with an acute investigation, as acute FMD following exercise may predict long-term FMD [13]. Our previous investigations [14,15] indicated that resistance exercise might acutely impair FMD in untrained young men. The higher blood pressure following exercise in untrained individuals may decrease FMD. Controlling blood pressure may be a critical factor in maintaining the intensity of resistance exercise while protecting against FMD impairment.

Combining elastic tubing with a free weight, which is currently used in conditioning programs [16,17,18,19], supplied the optimal degree of muscle load across the entirety of the range of motion (ROM). Consequently, the reduction in force during the first phase of ROM that causes the decrease in blood pressure may reduce the level of blood pressure to prevent the deterioration of vascular function, given that high blood pressure has been associated with a reduction in FMD [20]. The acute study by Paditsaeree and Mitranun [18] demonstrated that there was an increase in FMD between the baseline and after exercise parameters in the condition of combining elastic tubing with a free weight in untrained young adults. Nonetheless, under the condition of a free weight alone, FMD was reduced between the baseline and after exercise. Moreover, when comparing conditions, it was found that combining elastic tubing with a free weight resulted in greater FMD than that with the free weight alone. This combination program may be an interesting candidate for maintaining muscular function without exacerbating vascular impairment.

To the best of our knowledge, the acute effect of combined elastic and weight resistance training on endothelial function in older adults has not been investigated, particularly regarding different ranges of elastic and resistance modalities. Hence, we aimed to determine which combinations of elastic and weight resistance activities were the most effective for improving FMD and PWV in older adults. Therefore, this study aimed to examine the acute effects of the three exercise conditions on vascular function. 

## 2. Materials and Methods

### 2.1. Participants

Participants were recruited from a senior teacher club in Phuket, Thailand. The inclusion criteria were as follows: older adults aged 60–70 years; no participation in training programs for at least 6 months before the start of the study; no smoking; no vision, hearing, or vestibular functioning problems; no use of tranquilizers and antidepressants that affect balance; no unstable or ongoing respiratory/cardiovascular disorder; no musculoskeletal or neurological disease or impairment; and no history of injury within the previous year. The participants also completed a Senior Fitness Test (SFT); those with scores below the 25th percentile of age-based norms were excluded from the study because scores below the 25th percentile were interpreted as below average [21]. 

This study was approved by the Human Research Ethics Committee of Phuket Rajabhat University (No. PKRU2565/13) and was conducted in accordance with the guidelines of the Declaration of Helsinki. After a complete explanation of the exercise protocol and experimental procedures, all participants provided written informed consent.

Before the commencement of the exercise session, a Physical Activity Readiness Questionnaire Plus (PAR-Q+) was administered to assess the participants’ health state and readiness level. All participants were qualified to participate in the exercise sessions. The PAR-Q+ is used to discover previous medical issues that may be exacerbated by increased physical activity. Answering “no” to all seven questions on the PAR-Q+ provides reasonable certainty that the individual has a minimal risk of problems from low-to-moderate intensity exercise [22].

### 2.2. Experimental Design

**Figure 1:**
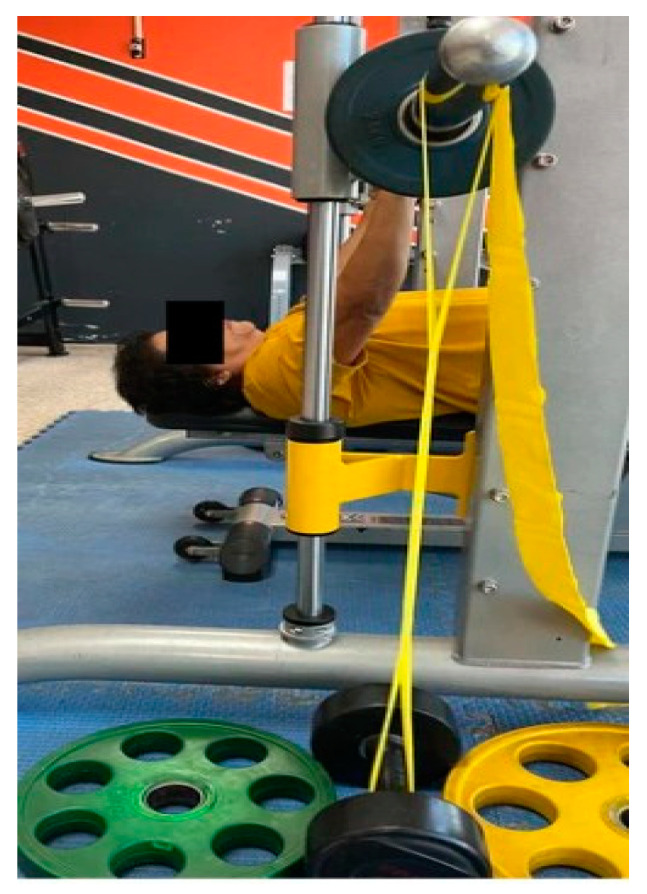
Setting the elastic bands on the barbell of a Smith machine for the bench press exercise.

This study was designed to evaluate three different resistance conditions for the Smith machine bench press (BP) exercise on FMD and PWV using a crossover design. In the first condition, the participants performed BP with the barbell alone (BA); in the second condition, they performed with a barbell combined with elastic bands at 10% of their 65% one repetition maximum (1 RM) (BE10); and in the third condition, they performed with a barbell combined with elastic bands at 20% of their 65% 1 RM (BE20). The elastic bands in the barbell of a Smith machine (Fex Fitness PTT0222, Fex Fitness Corp., Taiwan) used for the BP exercises are shown in Figure 1. We chose 65% of 1 RM because it is recommended in resistance training programs for older adults [23]. Each condition was separated for 72 h to the eliminate acute effects on FMD. The sequence of test conditions was selected using a 3 × 3 Latin square design.

Participants were required to attend the laboratory on five distinct occasions. Activities on day 1 (first day, Friday) comprised describing the test procedures, obtaining consent, collecting biometric data, and conducting the SFT. Day 2 (Monday) involved estimating 1 RM for the BP exercise, calculating resistance (65% of 1 RM), randomly separating three groups according to three types of resistance, and determining resistances for the BE10 and BE20 conditions. The three experimental conditions, scheduled for days 3, 4, and 5, were implemented one week following day 2. Friday, Monday, and Saturday were designated as Days 3, 4, and 5, respectively, to ensure participants had an ample recovery period between conditions. Each participant performed three sets of 12 repetitions of the BP exercise at 65% of 1 RM, with a 3 min rest period between sets to ensure sufficient recovery [23,24]. To ensure consistent repetition across all three sets, this study utilized 12 repetitions at 65% of 1 RM instead of 15 repetitions at 65% of 1 RM. This adjustment was made to prevent a decrease in the number of repetitions during sets 2 and 3. 

To standardize and regulate the intensity of the exercise across both conditions (BA and barbell with elastic bands), resistance was calculated based on the methodology outlined in a prior investigation by Wallace et al. [19]. This involved the following: (a) establishing the desired resistance value from the elastic bands (10% or 20% of the participants’ 65% 1 RM), and (b) deducting half of this value from the resistance provided by the barbell. The elastic bands were configured to apply the designated resistance value (10% or 20% of the participants’ 65% 1 RM) at the full contraction or the top phase of the BP exercise, when participants’ elbows were in close proximity rather than fully extended, while maintaining rigid wrists aligned directly over the elbows. Furthermore, the elastic bands began to exert tension when the barbell was in the bottom position of the lowering phase of the barbell BP when the bar was lowered until it touched the lower chest at approximately nipple level, with the upper arm abducted to approximately 45° at the shoulder joint. Additional forces of 10% and 20% from the elastic bands were chosen because these proportions fell within the range of values used in previous studies, representing a practical load exerted by elastic tension [18,25,26] and improved brachial FMD in untrained men [18]. 

The repetition duration for the BP was 3 s concentric and 4 s eccentric, as recommended for older adults [27]. The tempo was controlled using a metronome.

BP exercise was used in this study because it is a compound exercise for the upper body that strengthens the chest, shoulder, and triceps muscles. These muscles are located near the brachial arteries. The BP exercise was performed using a Smith machine to control balance throughout the ROM in older adults. The study indicates that bench press is a very effective exercise for enhancing the strength of the upper body in older adults [28]. Furthermore, the Smith machine bench press exercise was chosen because the tension of the elastic bands can be measured using a floor scale when the bands are extended in the Y-axis direction. In addition, the tension of elastic bands calculated in the linear direction from the direction of the bar of the Smith machine is more reliable than exercises such as dumbbell-side lateral raise that moves in a curved direction because the tension created by elastic bands in a linear direction remains constant when the bands extend by the same distance.

### 2.3. Experimental Procedures

#### Preliminary Testing

After clarifying the testing protocols and completing the informed consent process, all participants underwent the SFT [21] for at least 72 h before establishing their 1 RM BP. The SFT comprises the following six motor tests: back scratch, chair sit and reach, 30-s chair stand, 30-s arm curl, 2 min step, and 8-foot up-and-go testing. 

At least 72 h after completing the SFT, the participants performed a standardized warm-up session preceding the determination of their 1 RM of the BP. The warm-up comprised 5 min of stationary light cycling followed by 5 min of general stretching. After completing the warm-up session, the predicted 1 RM of each participant was determined under the supervision of a trained exercise physiologist. To measure 1 RM for the BP, the participants were tested for the maximum weight they could lift for no more than 4–8 repetitions (4–8 RM) of the BP, as this 4–8 RM range provides a reasonably accurate estimate of 1 RM [29]. Three to five attempts were required to identify the correct resistance [30]. A 5 min recovery period was provided between each failed attempt to ensure recovery [29]. This information was utilized to predict the 1 RM using the guidelines of the National Strength and Conditioning Associations [30] and compute the resistance (65% of the 1 RM) evaluated in this study. After determining 65% of the 1 RM of the BA conditions, the resistances of BE10 and BE20 were ascertained, as previously described in the experimental design.

After determining 65% of the 1 RM, 14 participants were randomly separated into three groups according to the three types of resistance. Therefore, each group executed all three experimental conditions on three separate days, with one condition per day.

### 2.4. Testing Procedures

The week after the preliminary tests, three different conditions (BA, BE10, and BE20) were tested. To provide sufficient recovery time for the participants, a 72 h period was chosen between testing sessions. Participants were instructed to abstain from exercise, alcohol, and over-the-counter medications for 24 h before the experimental testing session, and to avoid consuming coffee for 12 h. These variables have been found to alter flow-mediated vascular reactivity [31]. Additionally, the participants were advised to sleep sufficiently before the testing session. All of the tests were conducted simultaneously each day under controlled conditions (25 °C, 45–55% relative humidity).

Before collecting biometric, PWV, and FMD data, all participants were asked to fast, abstain from exercise, and avoid caffeine for 6 h to reduce the influence of confounding variables [32]. On the first day of assessing the three different conditions, the baseline values of the FMD, PWV, and biometric data were determined and used as a baseline for each subsequent day. Following the final set of BP tests, post-exercise evaluations were conducted immediately (0 min) for blood pressure, PWV, and FMD and 30 min later (30 min) for FMD. The participants’ biometric data included weight, height, body mass index, body fat percentage, resting heart rate, systolic blood pressure (SBP), diastolic blood pressure (DBP), and mean arterial pressure (MAP) values.

Body weight and fat content were recorded using a body composition analyzer (Tanita MC-780 MA, Tanita Corp., Tokyo, Japan). Before measuring blood pressure and heart rate (Carescape V100, GE Dinamap, GE Healthcare, Chicago, IL, USA), all participants rested in the supine position for at least 5 min.

After collecting blood pressure data, PWV data were examined using the approach of Yang et al. [33], in which PWV was measured using a volume plethysmographic device with four cuffs matched with oscillometric sensors positioned around the upper arms and ankles. During the deflation phase, pulse volumes of the bilateral brachial and tibial arteries were recorded. The blood pressure of each lesion was determined using an oscillometric technique. A microphone was attached to the left edge of the sternum, and the electrodes were positioned on both wrists. Transit time (Tba) was tracked using the time delay between the feet of waves at the two sites. The distance between the two PWV sites was determined using the participants’ height and ∆Tba. PWV was computed using the formula PWV = (La − Lb)/Tba, where Lb = 0.2195 × participant height (cm) − 2.0734 and La = 0.8129 × participant height (cm) + 12.328.

After measuring the PWV, FMD evaluations were performed according to a technique established by Corretti et al. [31] and Dhindsa et al. [34]. Brachial artery characteristic data were examined using the brachial artery occlusion of the right forearm and ultrasound equipment (Vivid i-GE Healthcare, Cardiovascular Ultrasound System; GE Medical Systems). To test FMD, all participants were instructed to rest comfortably in the supine position for 20 min, while a blood pressure cuff was attached to the right forearm throughout the 20 min period. The ultrasonography probe was positioned longitudinally above the antecubital fossa to image the brachial artery, and measurements were obtained at 1 min (baseline), 5 min of occlusion when the cuff was quickly inflated to 50 mmHg above the SBP, and 5 min of deflation. After deflating the cuff, the brachial artery diameter was recorded for 5 min. Using the pulsed-wave Doppler mode, the mean blood velocity was determined for all periods. In order to eliminate potential investigator bias in image analyses, a computer-based analysis software (Brachial Analyzer, Medical Imaging Applications, Coralville, IA, USA) was employed to assess alterations in vascular diameter. The following formula was used to obtain FMD data: FMD = (d2 − d1) × 100/d1, where d1 and d2 represent the average brachial artery diameter at baseline and after occlusion, respectively [35]. The FMD technique was executed by the same experienced operator who collected over 400 FMD data samples. In our laboratory, the intraclass correlation coefficient for FMD is >0.90. 

Before performing the BP, each participant performed a 5 min general cycling warm-up and a 5 min general stretching warm-up. The elastic band tensions in the BE10 and BE20 conditions were determined before the general warm-up to confirm that the band tension and condition were consistent with those on other testing days and that the bands were not damaged or deformed. Under the BE10 and BE20 conditions, elastic bands of three different sizes and colors—yellow, green, and blue (TheraBand CLX Consecutive Loops, TheraBand, Akron, OH, USA)—were used to generate resistance in conjunction with the barbell. Using two identical elastic bands and two identical dumbbells, both ends of the elastic bands were looped through dumbbell handles to the floor, whereas the other ends were hung from the end of the barbell and secured with clips. In addition, a floor scale (Defender 3000, Ohaus, Pine Books, NJ, USA) was used to determine the resistance of the three different colors or sizes of elastic bands to ensure that the elastic bands could produce both zero force and the prescribed force in the starting position and at full contraction of the BP exercise, respectively. 

After measuring the height from the ground to the starting position and full contraction of the BP exercise on a Smith machine for each participant, the bench was removed, and the floor scale was positioned in the middle of the Smith machine. The tension of the elastic bands was calculated using the difference between the weight of the individual standing on the floor scale with only the empty barbell handles of the Smith machine and the weight of the individual standing still on the floor scale with the end of the barbell attached to the elastic bands at the same height as that at the starting position and at full contraction during the BP exercise. 

For example, if 65% of the 1 RM was equal to 30 kg, then 10% of the participants’ 65% 1 RM would be calculated as 3 kg. Additionally, the height at which the elastic bands are stretched at the same height as that at full contraction during the BP exercise is 90 cm from the ground. Consequently, elastic bands that were stretched to a height of 90 cm could generate a force of 3 kg. If the weight of an individual standing on a floor scale while holding an empty barbell is 60 kg, then the weight of that individual standing still on the scale with the barbell’s ends attached to elastic bands that were stretched to a height of 90 cm could be displayed as 63 kg on the floor scale. This increase is due to the force exerted by the elastic bands, which adds an additional 3 kg of force (63 − 60 = 3). 

To determine 20% of the participants’ 65% 1 RM from the resistance of elastic bands, in the given example, if 65% of the 1 RM is equal to 30 kg, then 20% of the participants’ 65% 1 RM would be calculated as 6 kg. The elastic bands are stretched at the same height as those at full contraction during the BP exercise, which is 90 cm from the ground. Hence, elastic bands that were stretched to a height of 90 cm could generate a force of 6 kg. If the weight of an individual standing on a floor scale while holding an empty barbell is 60 kg, then the weight of that individual standing still on the scale with the barbell’s ends attached to elastic bands that were stretched to a height of 90 cm could be displayed as 66 kg on the floor scale. The increase is a result of the force exerted by the elastic bands, which provides an added 6 kg of force (66 − 60 = 6).

After setting up the machine, the participants performed three sets of 12 repetitions at 65% of the 1 RM. Furthermore, 3 min were allowed between the sets to ensure adequate recovery [23,24]. Warm-up sets were conducted progressively for the BP exercise before the execution of the work sets; however, 1–2 min rest periods were permitted between the warm-up sets.

### 2.5. Statistical Analysis

Values of each dependent variable and each condition are reported as the mean ± standard deviation (SD). To examine the interaction and main effects of time (pre-test [baseline], post-test [0 min], and post-test [30 min]) and condition while reporting the F- and *p*-values, a two-way (time × condition) repeated measures analysis of variance (ANOVA) was conducted. When necessary, the Bonferroni multiple comparison test was used to identify pairs of differences. Mauchly’s test was used to analyze the sphericity of the data. In cases where the sphericity assumption was violated, the Greenhouse–Geisser correction was applied. Moreover, partial eta-squared (η_p_^2^) was used to ascertain the effect size (ES) of the total treatment. The ES values interpreted for η_p_ were >0.01 (small), >0.06 (medium), and >0.14 (large) [36]. The ESs for contrasts were computed using an equation that converts F-values to r, where r is the square root of F(1, dfR)/(F(1, dfR + dfR) [37]. All statistical analyses were conducted using IBM SPSS Statistics for Windows, version 28.0 (IBM Corp., Armonk, NY, USA) with a significance level of *p* ≤ 0.05.

The percentage differences between each of the following conditions were calculated: baseline and BE20; baseline and BE10; baseline and BA; BA and BE20; BA and BE10; and BE20 and BE10. In addition, the percentage difference between 0 and 30 min after the BP exercise was calculated for each condition: BA (0 min) and BA (30 min), BE10 (0 min) and BE10 (30 min), and BE20 (0 min) and BE20 (30 min). An example of computing the percentage differences employed is shown in the following formula: ((BE10 − BA) × 100)/BA. Furthermore, the ESs between the baseline, BA, BE10, and BE20 conditions for each dependent variable were calculated using Cohen’s d_av_ statistic [38]. The magnitude of the ESs was calculated using the following scale: <0.50, 0.50–1.25, 1.25–1.9, and >2.0 for trivial, small, moderate, and large, respectively [39].

The sample size was similar to that of previous studies that involved 10 participants, who were required to distinguish a 2% change in the FMD percentage difference between the exercise conditions [40,41,42]. Furthermore, we assumed that the SD of this change was 2%, with 80% statistical power [41]. Based on this power estimate and to avoid dropouts, 14 participants were recruited for this study.

## 3. Results

This study examined a total of 14 participants. The baseline characteristics of participants are shown in Table 1. The means and SDs of the examined variables are presented in Table 1, Table 2 and Table 3, respectively. In addition, the mean and SD of the predicted 1-RM are 27.4 ± 12.0 kg. The mean resistance for the BA condition (65% 1 RM) is 17.8 ± 7.5 kg. In the BE10 condition, the mean resistance of the barbell is 16.9 ± 7.2 kg (65% of 1 RM minus half of the value from 10% of elastic bands), while the mean resistance of the elastic bands is 1.8 ± 0.8 kg (10% of 65% 1 RM). In the BE20 condition, the mean resistance of the barbell is 16.0 ± 6.8 kg (65% of 1 RM minus half of values from 20% of elastic band), while the mean resistance of the elastic bands is 3.6 ± 1.5 kg (20% of 65% 1 RM).

For the main effect of time for the peak FMD, the Greenhouse–Geisser estimate of the departure from sphericity was ԑ = 0.83. The two-way repeated measures ANOVA revealed no significant main effect of time on peak brachial diameter (PBD) (F[2,26] = 1.205, *p* = 0.316, η_p_^2^ = 0.085). The PBD was similar at baseline (mean = 4.74 mm), 0 min (mean = 4.73 mm), and 30 min (mean = 4.73 mm). The main effect of the condition on PBD was significant (F[2,26] = 4.24, *p* = 0.026, η_p_^2^ = 0.246). In contrast, the PBD of the BE10 condition (F(1,13) = 11.20, *r* = 0.68) was significantly greater than that of BA. 

For the interaction, the Greenhouse–Geisser estimate of the departure from sphericity was ε = 0.66. A significant interaction effect was found between the period of time and type of condition used (F(4,52) = 3.98). To break down this interaction, contrasts were used to compare all periods with their baseline and condition types to one another. These contrasts revealed significant interactions when comparing the BA condition to the BE10 condition for both 0 min compared with baseline (F[1,13] = 12.35, *p* = 0.004, *r* = 0.70) and 30 min compared with baseline (F[1,13]= 6.41, *p* = 0.025, *r* = 0.57).

The Greenhouse–Geisser estimate of the departure from sphericity for the main effect of time for FMD was ԑ = 0.82. The two-way repeated measures ANOVA revealed no significant main effect of time on FMD (F[2,26] = 1.123, *p* = 0.341, η_p_^2^ = 0.079). The FMD was identical at baseline (mean = 5.81%), 0 min (mean = 5.53%), and 30 min (mean = 5.56%). A significant main effect of condition was found on FMD, as shown by the values F(2,26) = 3.86, *p* = 0.034, and η_p_^2^ = 0.229. In contrast, the FMD of the BE10 condition was significantly greater than that of the BA condition (F[1,13] = 10.35 and *r* = 0.67). The Greenhouse–Geisser estimate of the divergence from sphericity for the interaction was ε = 0.67. A significant interaction effect (F[4,52] = 3.66) was observed between the period of time and type of condition employed. To dissect this interaction using contrast, all periods and condition types were compared to their baseline values. These contrasts showed that there were significant interactions when comparing the BA condition to the BE10 condition, both for 0 min compared to baseline (F[1,13] = 12.85, *p* = 0.003, *r* = 0.71) and for 30 min compared to baseline (F[1,13] = 5.12, *p* = 0.041, *r* = 0.53).

**Figure 2:**
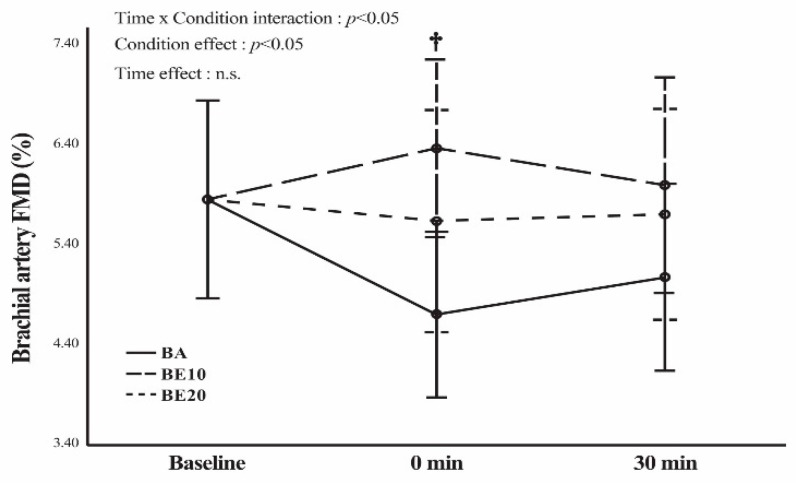
Brachial artery %flow-mediated dilation (FMD) in barbell alone (BA), barbell combining elastic bands at 10% of the participants’ 65% 1 RM (BE 10), and barbell combining elastic bands at 20% of the participants’ 65% 1 RM (BE 20). Data are expressed as means and 95% confidence interval error bars. A two-way (time × group) repeated measures analysis of variance (ANOVA) was performed. † *p* < 0.05 for the difference between BE10 and BA. n.s. = not significant (*p* > 0.05).

Using pairwise comparisons, the statistical analysis of FMD and PBD at 0 min after BP exercise showed a statistically significant difference (*p* < 0.05) between the BE10 and BA conditions (Figure 2 and Table 2). No significant difference (*p* > 0.05) was observed 30 min after the BP exercise among the three conditions. Moreover, the percentage changes and ESs indicated that BE10 had a greater treatment effect than did BA (Table 4 and Table 5). No significant difference was found in FMD and PBD between baseline and 0 min after exercise in all conditions (*p* ≥ 0.05), as shown in Figure 2 and Table 2. The percentage changes and ESs for all variables are presented in Table 4 and Table 5, respectively. No significant difference was observed in all conditions (*p* ≥ 0.05) between baseline and 30 min after exercise in each condition for FMD and PBD, as shown in Figure 2 and Table 2. The percentage changes and ESs for all variables are presented in Table 6 and Table 7, respectively. In addition, no significant differences were found in FMD and PBD between 0 and 30 min after exercise under any condition (*p* < 0.05) (Figure 2 and Table 2). The percentage changes and ESs for all variables are presented in Table 8 and Table 9, respectively.

For the main effect of the condition on PWV, the Greenhouse–Geisser estimate of the departure from sphericity was ԑ = 0.99. The outcomes of the two-way repeated-measures ANOVA revealed that the main effect of the condition on PWV was statistically significant: F(2,26) = 4.51, *p* = 0.021, and η_p_^2^ = 0.257. The contrasts demonstrated that the PWV of the BA condition was significantly higher than that of the BE10 condition with F(1,13) = 8.13 and *r* = 0.62.

**Figure 3:**
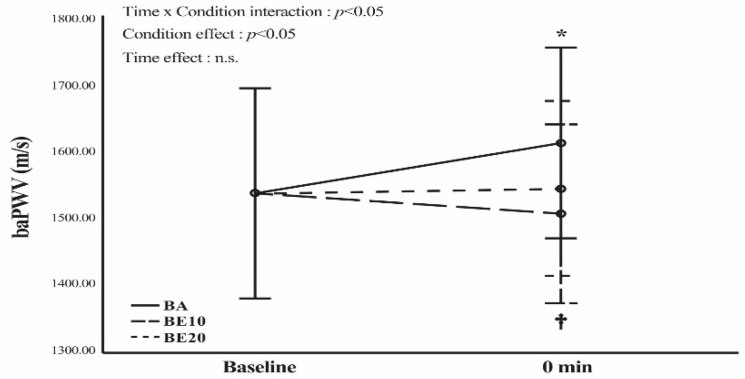
Brachial-ankle pulse wave velocity (baPWV) in barbell alone (BA), barbell combining elastic bands at 10% of the participants’ 65% 1 RM (BE10), and barbell combining elastic bands at 20% of the participants’ 65% 1 RM (BE20) conditions. Data are expressed as means and 95% confidence interval error bars. A two-way (time × group) repeated measures analysis of variance (ANOVA) was performed. * *p* < 0.05 for the difference from the baseline value in BA. † *p* < 0.05 for the difference between BA and BE10. n.s. = not significant (*p* > 0.05).

Regarding the PWV data at 0 min after the BP exercise, using a pairwise comparison, a significant difference was found between the BA and BE10 conditions (*p* < 0.05). However, no significant differences were observed between the BA and BE20 conditions (*p* < 0.05) or between the BE10 and BE20 conditions (*p* < 0.05) (Figure 3). In addition, as shown in Figure 3, the statistical analysis showed a significant increase (*p* < 0.05) in PWV compared to the baseline value only in the BA condition. As presented in Table 4 and Table 5, the percentage changes and ESs demonstrated a greater treatment effect in the BA condition than in the baseline condition.

**Figure 4:**
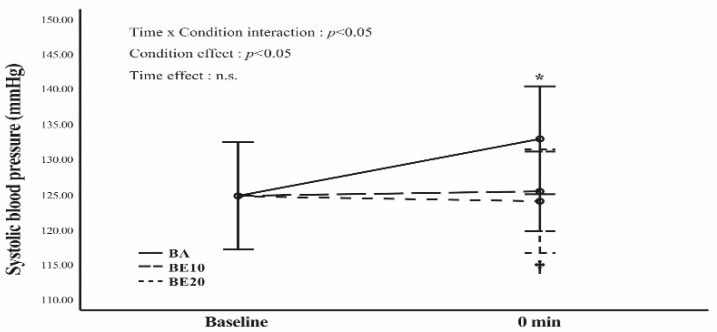
Systolic blood pressure before and after resistance exercise in barbell alone (BA), barbell combining elastic bands at 10% of the participants’ 65% 1 RM (BE10), and barbell combining elastic bands at 20% of the participants’ 65% 1 RM (BE20) conditions. Data are expressed as means and 95% confidence interval error bars. A two-way (time × condition) repeated measures analysis of variance (ANOVA) was performed. * *p* < 0.05 for the difference from the baseline value in BA. † *p* < 0.05 for the difference between BA and BE20. n.s. = not significant (*p* > 0.05).

Regarding the main effect of the condition on SBP, the Greenhouse–Geisser estimate of the departure from sphericity was ԑ = 0.82. A significant main effect of the condition was found on SBP, as represented by F(2,26) = 5.06, *p* = 0.014, and η_p_^2^ = 0.280. The SBP in the BA condition (F[1,13] = 14.75, *r* = 0.73) was significantly higher than that in the BE20 condition. Regarding SBP using the paired comparison method, SBP at 0 min after exercise was significantly higher in the BA condition than in the BE20 (*p* < 0.05). For the post-exercise state, only the BA condition showed a significant increase in SBP compared to the pre-exercise state (*p* < 0.05), as shown in Figure 4.

Based on the main effect of the condition on DBP, the Greenhouse–Geisser estimate of the departure from sphericity was ԑ = 0.94. The main effect of the condition on DBP was not statistically significant (F[2,26] = 0.46, *p* = 0.635, η_p_^2^ = 0.034). Moreover, for the main effect of the condition on MAP, the Greenhouse–Geisser estimate of the departure from sphericity was ԑ = 0.89. The main effect of the condition on MAP was not significant (F[2,26]= 1.34, *p* = 0.278, η_p_^2^ = 0.094).

Regarding MAP and DBP using pairwise comparison, no significant differences (*p* ≥ 0.05) were observed at 0 min after BP exercise among the three conditions (Table 3). In addition, no differences (*p* ≥ 0.05) were found between baseline and post-exercise blood pressure measurements (MAP and DBP) in any of the three conditions (BA, BE10, and BE20).

## 4. Discussion

The major finding of this study was that the FMD of the BA10 condition was significantly greater than that of the BA at 0 min after exercise. When comparing the FMD at baseline, 0 min, and 30 min after exercise, it remained unchanged in all conditions. In addition, our data revealed a significant increase in PWV at 0 min after exercise compared to the baseline value only in the BA condition. At 0 min post-exercise, the BA condition also had a significantly higher PWV than that in the BE10 condition.

The immediate impacts of resistance exercise on FMD depend on several variables [43], including intensity [44], the rate of muscle contraction [20], diverse training postures [14,45], and the fitness level of participants [46]. To mitigate the influence of these potential confounding variables, we selected a BP posture that is recommended for older adults. Furthermore, the durations of both the concentric and eccentric phases were standardized across all three groups at 3 and 4 s, respectively, to negate the effects of muscle contraction velocity [20]

Regarding ROM, the force outputs of the three interventions, BA, BE10, and BE20, were distinct. The BA condition is a resistance training pattern that produces the greatest torque at the beginning of the ROM and gradually decreases near the end of the ROM. BE10 and BE20 are variable resistances that combine a barbell and an elastic band to generate equal force across the full ROM by reducing deceleration at the end of the concentric phase [19]. Moreover, the elastic bands provide the greatest external load at the end of the concentric phase owing to the increased stretching of the elastic tubing [47]. To understand the influence of these three interventions, brachial characteristic responses were measured at baseline, 0 min, and 30 min after exercise.

The BA condition demonstrated no change in FMD from baseline to 0 min and from baseline to 30 min post-exercise. However, the statistical findings did not reach significance and revealed decreased percentages of change in FMD (19.82% and 13.35%, respectively). These findings are consistent with those of previous studies that investigated the following exercises: leg extensions with three sets of eight repetitions at 50% of 1 RM [48], where FMD decreased from baseline to 10 and 30 min post-exercise (1.03% and 7.85%, respectively) and leg press exercises of 6–8 repetitions for 2–3 sets [46], where the FMD decreased from baseline to post-exercise (28.75%). Variations in blood pressure may account for these outcomes. High blood pressure is associated with decreased FMD because of its vasoconstrictive effects [20]. Prolonged elevated blood pressure may inhibit NO, an endothelial vasodilator [49]. BA is a strong intervention that causes significantly greater increases in SBP, DBP, and MAP than do BE10 and BE20. Another reason is that a crucial mechanism for the vasoconstrictive effect has been identified through an increase in sympathetic activity during resistance exercises, particularly upper-body resistance exercises [10]. These were our justifications for the lowest FMD in the BA.

In the BE20 condition, no change was found in FMD, and statistical analysis revealed a slight reduction in blood pressure compared with the baseline value. This outcome may be explained by the fact that when combined with the intensity of training with BA, BE20, which generates the lowest torque at the initial ROM of concentric motion, may lessen the negative effects of resistance training. Increased sympathetic nervous activity (SNA) is another potential mechanism associated with this outcome. In fact, SNA activation is associated with impaired FMD, regardless of the presence [44] or absence [50] of exercise intervention. During resistance training, an increase in the SNA resulted from both concentric and eccentric motions [51]. We did not assess the impact of SNA; however, we assume that SNA activity in the BE20 is lower than that in the BA because of the different patterns of force generation described above.

Remarkably, while it could not reach statistical significance, only the BE10 condition revealed an increase in FMD after the BP exercise. Furthermore, the FMD in BE10 was significantly greater than that in BA after 0 min of exercise. Blood pressure did not change in BE10 after exercise and was lower in BE10 than in BA. The vasodilatory effects appear to be preferable to the vasoconstrictive effects that BE10 may elicit by immediately enhancing vascular function. BE10 combines the patterns of weight resistance and elastic resistance at 10%. Therefore, the force distribution was more consistent, and the peak force may be lower for BE10 than for BA [52] and BE20, which generated a greater torque near the end of the ROM for concentric motion. Possibly, these effects may diminish SNA activation. Additional research is needed to explore the significant variation in FMD observed specifically in BE10 as opposed to BE20. This investigation should aim to uncover potential mechanisms such as oxidative stress, vasodilators, vasoconstrictors, and others [43], in order to provide a comprehensive understanding of the distinct phenomena observed between BE10 and BE20.

Regarding the brachial-ankle-PWV in the BA condition, the increase from baseline to 0 min after exercise was 4.97%. This outcome was similar to those of previous studies, which indicated a transitory increase in arterial stiffness after acute resistance exercise [53,54]. Heffernan et al. [53] reported a significant increase of 20.8% from baseline to 20 min in carotid-femoral-PWV (cf-PWV) following an acute session of resistance exercise when participants performed three sets of 10 repetitions (executed at 100% of the participants’ 10 RM) of eight exercises. Furthermore, Kingsley et al. [54] discovered a significant increase of 9.6% in cf-PWV from baseline to 10 min post-exercise when participants completed three sets of 10 repetitions at 75% of 1 RM for squat, BP, and deadlift exercises. These outcomes may be explained by the fact that traditional resistance training induces highly elevated SBP and DBP due to the mechanical compression of blood vessels with each contraction, along with a strong exercise pressor reflex and Valsalva response [55]. Consequently, elevated blood pressure in the BA condition may cause a transitory switch in load-bearing from elastin to collagen fibers in the arteries, leading to an increase in PWV [55]. In contrast, blood pressure was lower in the BE10 and BE20 conditions than in the BA condition because the BE10 and BE20 conditions created less torque during the initial ROM of the concentric motion. Hence, the PWV values after acute exercise in the BE10 and BE20 conditions did not significantly increase from baseline.

This study had some limitations. First, the sample size was relatively small. Second, smooth muscle function was not evaluated in this study, which might have helped to elucidate the alterations that occurred in FMD and PWV. Third, the gender distribution in this study is imbalanced, with six males and eight females. Subsequent research may wish to explore the impact of sex differences. Fourth, only brachial characteristic responses were examined in this study. Therefore, future research should include other vascular areas and variables, such as sympathetic nervous system activity, to better understand the underlying mechanisms. Furthermore, further study should be conducted on older adults who have different levels of experience with exercise training. This is due to the observation that vascular function can be altered following acute exercise, depending on the training status of young male participants [56].

## 5. Conclusions

After exercise, resistance training alone had a greater detrimental effect on FMD than resistance training combined with 10% elastic resistance. Additionally, weight training alone had immediate negative effects on PWV. However, PWV was unaffected by the combination of weight and elastic resistance. Furthermore, weight training alone produced more immediate negative effects on PWV than the combination of weight training and elastic resistance at 10%. When analyzing the effects of combining elastic and weight resistance, we surprisingly discovered that combinations of weight and elastic resistance at 10% may be more effective in preventing a decrease in FMD and an increase in PWV than combinations of weight and elastic resistance at 20%. Consequently, BA, BE10, and BE20 exhibited diverse modifications in vascular function. These findings need to be validated through long-term training intervention studies.

## Figures and Tables

**Table 1:** Baseline characteristics of participants.

Characteristics (N = 14, Male = 6)	Mean ± SD
Age (y)	65.6 ± 2.9
Body mass (kg)	63.4 ± 14.1
Height (cm)	159.8 ± 8.5
Body mass index (kg/m^2^)	24.6 ± 4.4
Body fat (%)	30.6 ± 7.8
Number of underlying disease (hypertension, dyslipidemia)	3 (21.4%)

SD, standard deviation.

**Table 2:** Mean ± SD values for brachial characteristics before and after the BP exercise in each condition (N = 14) *.

Dependent Variable		Baseline	0 min	30 min	ANOVA
	BA	4.74 ± 0.75	4.68 ± 0.71	4.70 ± 0.74	Interaction: *p* < 0.05
Peak brachial diameter (mm)	BE10	4.74 ± 0.75	4.76 ± 0.75 †	4.75 ± 0.76	Condition: *p* < 0.05
	BE20	4.74 ± 0.75	4.73 ± 0.74	4.73 ± 0.74	Time: n.s.

* BL = baseline; BA = a barbell alone; BE10 = a barbell combining elastic bands at 10%; BE20 = a barbell combining elastic bands at 20%; SD = standard deviation; ANOVA = analysis of variance; BP = bench press. † *p* < 0.05 vs. BA; n.s. = not significant (*p* > 0.05).

**Table 3:** Mean ± SD values for diastolic blood pressure and mean arterial pressure before and at 0 min after the BP exercise in each condition (N = 14) *.

Dependent Variable		Baseline	0 min	ANOVA
	BA	72.8 ± 7.2	76.1 ± 7.8	Interaction: n.s.
Diastolic blood pressure (mmHg)	BE10	72.8 ± 7.2	74.3 ± 7.5	Condition: n.s.
	BE 20	72.8 ± 7.2	74.2 ± 8.8	Time: n.s.
	BA	94.8 ± 10.3	98.6 ± 9.9	Interaction: n.s.
Mean arterial pressure (mmHg)	BE10	94.8 ± 10.3	95.1 ± 9.2	Condition: n.s.
	BE20	94.8 ± 10.3	94.4 ± 11.0	Time: n.s.

* BL = baseline; BA = a barbell alone; BE10 = a barbell combining elastic bands at 10%; BE20 = a barbell combining elastic bands at 20%; SD = standard deviation; ANOVA = analysis of variance; BP = bench press; n.s. = not significant (*p* > 0.05).

**Table 4:** Percentage changes among baseline, BE20, BE10, and BA conditions at 0 min after the BP exercise for each dependent variable *.

Dependent Variable	Percentage Change					
	BL to BE20	BL to BE10	BL to BA	BA to BE20	BA to BE10	BE20 to BE10
Flow-mediate dilatation (%)	−3.70	8.89	−19.82	20.10	35.81	13.08
Peak brachial diameter (mm)	−0.21	0.48	−1.18	0.98	1.68	0.69
Pulse wave velocity (m/s)	0.43	−2.02	4.97	−4.32	−6.66	−2.45
Mean arterial pressure (mmHg)	−0.45	0.30	3.99	−4.28	−3.55	0.76
Systolic blood pressure (mmHg)	−0.57	0.57	6.48	−6.62	−5.55	1.15
Diastolic blood pressure (mmHg)	1.96	2.06	4.51	−2.44	−2.35	0.10

* BL = baseline; BA = a barbell alone; BE10 = a barbell combining elastic bands at 10%; BE20 = a barbell combining elastic bands at 20%; BP = bench press.

**Table 5:** Effect sizes among baseline, BE20, BE10, and BA conditions at 0 min after the BP exercise for each dependent variable *.

Dependent Variable	Effect Sizes					
	BE20 and BL	BE10 and BL	BA and BL	BE20 and BA	BE10 and BA	BE10 and BE20
Flow-mediated dilatation (%)	−0.12	0.32	−0.73	0.56	1.12	0.42
Peak brachial diameter (mm)	−0.01	0.03	−0.08	0.06	0.11	0.04
Pulse wave velocity (m/s)	0.03	−0.12	0.29	−0.29	−0.45	−0.16
Mean arterial pressure (mmHg)	−0.04	0.03	0.38	−0.40	−0.37	0.07
Systolic blood pressure (mmHg)	−0.05	0.06	0.61	−0.68	−0.64	0.13
Diastolic blood pressure (mmHg)	0.18	0.20	0.44	−0.22	−0.23	0.01

* BL = baseline; BA = a barbell alone; BE10 = a barbell combining elastic bands at 10%; BE20 = a barbell combining elastic bands at 20%; BP = bench press.

**Table 6:** Percentage changes among baseline, BE20, BE10, and BA conditions at 30 min after the BP exercise for FMD and PBD variables *.

Dependent Variable	Percentage Change					
	BL to BE20	BL to BE 10	BL to BA	BA to BE 20	BA to BE 10	BE 20 to BE 10
FMD (%)	−2.53	2.49	−13.35	12.49	18.29	4.90
PBD (mm)	−0.15	0.17	−0.75	0.61	0.93	0.32

* BL = baseline; BA = a barbell alone; BE10 = a barbell combining elastic bands at 10%; BE20 = a barbell combining elastic bands at 20%; BP = bench press; FMD = flow-mediated dilation; PBD = peak brachial diameter.

**Table 7:** Effect sizes among baseline, BE20, BE10, and BA conditions at 30 min after the BP exercise for FMD and PBD variables *.

Dependent Variable	Effect Sizes					
	BE20 and BL	BE10 and BL	BA and BL	BE20 and BA	BE10 and BA	BE10 and BE20
FMD (%)	−0.08	0.08	−0.46	0.36	0.53	0.16
PBD (mm)	−0.01	0.01	−0.05	0.04	0.06	0.02

* BL = baseline; BA = a barbell alone; BE10 = a barbell combining elastic bands at 10%; BE20 = a barbell combining elastic bands at 20%; BP = bench press; FMD = flow-mediated dilation; PBD = peak brachial diameter.

**Table 8:** Percentage changes among BE20, BE10, and BA conditions at 0 min and 30 min after the BP exercise for FMD and PBD variables *.

Dependent Variable	Percentage Change		
	BA (0 min) to BA (30 min)	BE10 (0 min) to BE10 (30 min)	BE20 (0 min) to BE20 (30 min)
FMD (%)	8.06	−5.88	1.21
PBD (mm)	0.43	−0.31	0.06

* BL = baseline; BA = a barbell alone; BE10 = a barbell combining elastic bands at 10%; BE20 = a barbell combining elastic bands at 20%; BP = bench press; FMD = flow-mediated dilation; PBD = peak brachial diameter.

**Table 9:** Effect sizes among BE20, BE10, and BA conditions at 0 min and 30 min after the BP exercise for FMD and PBD variables *.

Dependent Variable	Effect Sizes		
	BA (30 min) and BA(0 min)	BE10 (30 min) and BE10 (0 min)	BE20 (30 min) and BE20 (0 min)
FMD (%)	0.25	−0.22	0.04
PBD (mm)	0.03	−0.02	0.00

* BL = baseline; BA = a barbell alone; BE10 = a barbell combining elastic bands at 10%; BE20 = a barbell combining elastic bands at 20%; BP = bench press; FMD = flow-mediated dilation; PBD = peak brachial diameter.

## Data Availability

Available upon request.
